# Topological protection versus degree of entanglement of two-photon light in photonic topological insulators

**DOI:** 10.1038/s41467-021-22264-3

**Published:** 2021-03-30

**Authors:** Konrad Tschernig, Álvaro Jimenez-Galán, Demetrios N. Christodoulides, Misha Ivanov, Kurt Busch, Miguel A. Bandres, Armando Perez-Leija

**Affiliations:** 1 Max-Born-Institut, Berlin, Germany; 2 Humboldt-Universität zu Berlin, Institut für Physik, AG Theoretische Optik & Photonik, Berlin, Germany; 3 CREOL, The College of Optics and Photonics, University of Central Florida, Orlando, FL USA; 4 Blackett Laboratory, Imperial College London, London, UK

**Keywords:** Single photons and quantum effects, Quantum information

## Abstract

Topological insulators combine insulating properties in the bulk with scattering-free transport along edges, supporting dissipationless unidirectional energy and information flow even in the presence of defects and disorder. The feasibility of engineering quantum Hamiltonians with photonic tools, combined with the availability of entangled photons, raises the intriguing possibility of employing topologically protected entangled states in optical quantum computing and information processing. However, while two-photon states built as a product of two topologically protected single-photon states inherit full protection from their single-photon “parents”, a high degree of non-separability may lead to rapid deterioration of the two-photon states after propagation through disorder. In this work, we identify physical mechanisms which contribute to the vulnerability of entangled states in topological photonic lattices. Further, we show that in order to maximize entanglement without sacrificing topological protection, the joint spectral correlation map of two-photon states must fit inside a well-defined topological window of protection.

## Abstract (web summary)

Topological protection of entangled states is a promising avenue for photonic quantum technologies. Here, Tschernig et al. theoretically analyse the impact of disorder on topological protection of entangled two-photon states in periodic and aperiodic topological insulator lattices.

## Introduction

The prospect of generating topologically protected entangled states of several photons is a highly intriguing proposition^1–3^. Specifically, topological protection can enable robust transport of quantum information across disordered photonic structures without degradation^4,5^, just as efficiently as for single-particle wavepackets^6–10^.

In recent years, we have witnessed several experimental demonstrations of topological protection at the single-photon level in integrated one-dimensional lattice systems. Notably, Wang and co-workers^11^ showed that the fundamental quantum features of spatially entangled biphoton-states can be protected against disorder in the so-called Su-Schrieffer-Heeger (SSH) topological lattice. Interestingly, SSH lattices turned out to be equally effective in protecting polarization-entangled photon pairs^12^. Another important ingredient was provided by Tambasco et al.^13^ showing that Hong-Ou-Mandel two-photon interference of topological edge–modes is feasible, by implementing a topological beamsplitter in a judiciously engineered time-dependent Harper-model.

Concurrently, on the theory front several ideas have been suggested to investigate topological two-photon effects in linear^14,15^ and nonlinear^16^ lattice systems. In this regard, an intriguing proposition was recently put forward^17^, where the Bose-Hubbard model, which is topologically trivial for single particles, becomes topologically nontrivial for two interacting photons. That is, particle interactions have a dramatic impact on topological properties, not only modifying the topology of the spectra but also creating a topological order in otherwise topologically trivial systems.

In order to maximize the potential of topological photonic networks for transferring quantum information, it is indispensable to have a considerable number of edge modes at our disposal. One possibility is to use two-dimensional topological systems, which intrinsically support a multitude of topological edge-states^18–20^.

In two-dimensional photonic topologial insulators, single-particle edge-states reside in the gap existing between the energy bands supporting the bulk states^21–23^. Thus, breaking the topological protection requires disorder with sufficient strength to close the bandgap. For states describing two indistinguishable photons, the same bandgap is fundamentally lacking. The reason is because the propagation eigenvalues ${\lambda }_{12}^{(2)}$ for two-photon eigenstates in a photonic system are given by the sum of the eigenvalues *λ*_1_, *λ*_2_ corresponding to the constituent individual photons, ${\lambda }_{12}^{(2)}={\lambda }_{1}+{\lambda }_{2}$. This implies that we can keep ${\lambda }_{12}^{(2)}$ constant while increasing *λ*_1_ and simultaneously decreasing *λ*_2_, or vice versa. In this way, we can combine two single-photon bulk states, one from the lower and one from the upper band, to create a biphoton bulk–bulk state whose energy lies inside the single-particle bandgap. This fundamental additive property of the single-particle eigenvalues removes the bandgap and leads to massive degeneracies of the edge–edge, edge–bulk, and bulk–bulk two-photon states. Hence, considering the lack of the topological bandgap for two-photon systems, it is not clear whether topological protection will be automatically granted to two-particle states provided single particles are topologically protected in the same system.

In solids, the degeneracies described above lead to the decay of two-electron edge states when electron–electron correlations are substantial^24–26^. This decay mechanism is reminiscent of auto-ionization, where electron–electron correlations lead to energy exchange between the two particles, coupling two bound electronic excitations to an energy-degenerate bound-continuum two-electron state^27,28^.

Still, photonic systems are fundamentally different from solids, as the two photons do not readily interact with each other^29^. Consequently, the evolution operator for two-photon states, *U*^(2)^(*z*), breaks down into the product of two propagators for individual single-photon states, *U*^(2)^(*z*) = *U*(*z*) ⊗ *U*(*z*)^30^. Thus, a natural question to ask is whether such a factorization and the absence of bangap will prevent decoherence and dissipation of non-factorizable two-photon edge-states into the bulk?

In this work, we analyze possible mechanisms of dissipation of two-photon edge states into the bulk of two different topological insulator system, the Haldane lattice model and an aperiodic lattice corresponding to the quantum Hall effect. Our results show that the key to topological protection is to minimize the disorder-induced overlap of the initial two-photon (joint) spectrum with the edge–bulk and bulk–bulk spectral regions.

## Results

### Theoretical approach

In lattice systems, static disorder can be introduced in either the site energies—termed diagonal disorder^31^—or in the coupling coefficients—so-called off-diagonal disorder^32^. In either case, static disorder is represented by a single-particle operator ${\hat{V}}^{(1)}$. Since such perturbation is time-independent, energy conserving resonant coupling into the bulk is absent within first-order perturbation theory—the single-particle transition induced by ${\hat{V}}^{(1)}$ does not preserve energy. The process that can resonantly couple a two-photon edge–edge state to a bulk–bulk, or to a bulk–edge, state would require a correlated change of states for both photons and it might arise within the second-order corrections in ${\hat{V}}^{(1)}$.

To see this, we examine the second-order transition matrix elements between an initial two-photon edge–edge state $\left|{\rm{i}}\right\rangle =\left|{n}_{{\rm{i}}},{m}_{{\rm{i}}}\right\rangle$ and a final edge–bulk, or bulk–bulk, state $\left|{\rm{f}}\right\rangle =\left|{n}_{{\rm{f}}},{m}_{{\rm{f}}}\right\rangle$

1 $${V}_{{\rm{i}},{\rm{f}}}^{(2)}=\mathop{\sum} \limits_{j^{\prime} }\frac{{V}_{{\rm{f}}j^{\prime} }^{(1)}{V}_{j^{\prime} {\rm{i}}}^{(1)}}{{\lambda }_{j^{\prime} }^{(2)}-{\lambda }_{{\rm{i}}}^{(2)}},$$

where $\left|j^{\prime} \right\rangle =\left|n^{\prime} ,m^{\prime} \right\rangle$ labels intermediate *virtual states* and ${\lambda }_{j^{\prime} }^{(2)}={\lambda }_{n^{\prime} }+{\lambda }_{m^{\prime} }$. The single-particle nature of ${\hat{V}}^{(1)}$ ensures that only two terms corresponding to the two possible time-orderings of the two single-particle transitions are left in the sum

2 $${V}_{{\rm{i}},{\rm{f}}}^{(2)}={V}_{{n}_{{\rm{f}}},{n}_{{\rm{i}}}}^{(1)}{V}_{{m}_{{\rm{f}}},{m}_{{\rm{i}}}}^{(1)}\left[\frac{1}{{\lambda }_{{n}_{{\rm{f}}}}-{\lambda }_{{n}_{{\rm{i}}}}}+\frac{1}{{\lambda }_{{m}_{{\rm{f}}}}-{\lambda }_{{m}_{{\rm{i}}}}}\right].$$

The stationary nature of the disorder dictates that real transitions from the edge–edge states can occur only if the initial eigenvalue ${\lambda }_{{\rm{i}}}^{(2)}={\lambda }_{{n}_{{\rm{i}}}}+{\lambda }_{{m}_{{\rm{i}}}}$ is equal to the final one ${\lambda }_{{\rm{f}}}^{(2)}={\lambda }_{{n}_{{\rm{f}}}}+{\lambda }_{{m}_{{\rm{f}}}}$. Therefore, ${\lambda }_{{n}_{{\rm{f}}}}-{\lambda }_{{n}_{{\rm{i}}}}=-({\lambda }_{{m}_{{\rm{f}}}}-{\lambda }_{{m}_{{\rm{i}}}})$, and the two terms in ${V}_{{\rm{i}},{\rm{f}}}^{(2)}$ exactly cancel each other, ${V}_{{\rm{i}},{\rm{f}}}^{(2)}=0$. That is, two-particle dissipation from each product state is zero, and the same is true for any entangled two-particle state *ψ*^(2)^ represented as a quantum superposition of two possible distinguishable configurations ${\psi }_{{\rm{i}}}^{(2)}$, e.g., ${\psi }^{(2)}={\psi }_{1}^{(2)}+{\psi }_{2}^{(2)}$. Physically, this destructive interference is a direct outcome of the indistinguishability of the photons, which ensures that the two-particle eigenvalue is a sum of the two single-particle eigenvalues, and that the two-particle propagator is a product of the single-particle counterparts.

In contrast to dissipation, the situation with dephasing can be different: while each constituent state in an entangled superposition can be protected against disorder^1^, the overall superposition is, in general, not. To be precise, motion through different disordered regions may lead to disorder-induced random phase shifts between the states destroying the entanglement. To avoid this fate, all states in the superposition must travel across the same spatial region of the photonic structure, such that they are affected by disorder in the exact same manner^33^. These effects have been explored for spatial path-entangled states^1^, and for states built from an entangled superposition of an initial non-stationary state ${\psi }_{1}^{(2)}$ with its time-delayed replica ${\psi }_{1}^{(2)}(\tau )$, that is, entangled states in the time-domain^2^. In these two cases, however, the entanglement of the states can be related to the entanglement of symmetrized wavefunction of identical particles^34^. Consequently, the states exhibit the lowest possible amount of entanglement, as indicated by the corresponding Schmidt numbers *S*_N_ = 2. Throughout this work we use the Schmidt number to quantify the amount of entanglement: *S*_N_ = 1 denotes complete separability while *S*_N_ ≫ 1 corresponds to high entanglement^35^.

A more appealing type of highly entangled two-photon states are multimode optical Gaussian states in which both photons are most likely to be found inhabiting any waveguide, within an excitation window, simultaneously^36^. The importance of such states is based on the fact that any phase difference arising among the paths becomes enhanced by a factor of two in comparison with single photon states^37^. Naturally, the enhanced phase sensitivity of such highly entangled two-photon states manifests as faster diffraction of the associated wavepackets propagating in any photonic system, periodic and disordered^38^. Therefore, it is not clear to what extent topological protection will persist for these types of highly entangled states.

### Propagation of entangled two-photon states in disordered topological lattices

In what follows we analyze the impact of disorder onto a continuum of two-photon states that extend from the correlated to the anti-correlated limits, passing through a completely separable state. For our analysis we consider two topological lattices, one periodic and one aperiodic. In the periodic case we consider the Haldane model^39^, and for the aperiodic we use a square lattice whose single-particle dynamics corresponds to the quantum Hall effect^6,40^ (information, S. Supporting material). The results for the Haldane model are presented here, while the quantum Hall effect lattice is discussed in the Supplementary Note 5.

**Fig. 1:**
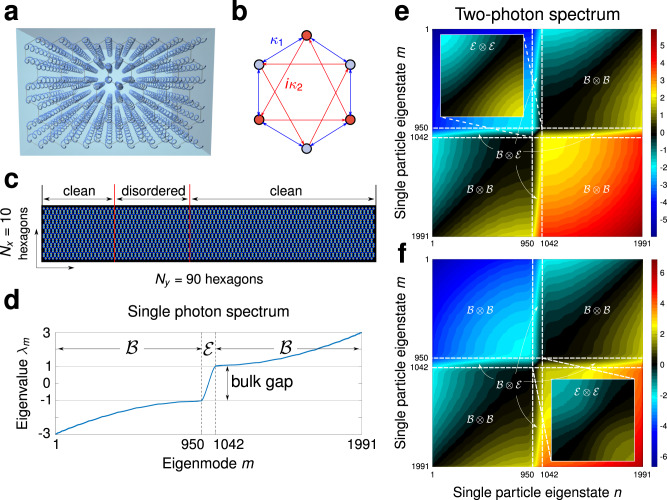
The Haldane photonic lattice. **a** Photonic implementation of the Haldane system using a honeycomb lattice of helical waveguides. **b** Elementary hexagonal cell of the Haldane system, with real-valued nearest-neighbor coupling (blue arrows) *κ*_1_ = 1 and imaginary next-nearest-neighbor coupling (red arrows); *κ*_2_ = *iκ*_1_/5 along the arrow and − *iκ*_1_/5 in the opposite direction. **c** Pictorial view of the finite lattice used in our numerical analysis. **d** Single-photon spectrum formed by eigenvalues *λ*_*n*_. In **e** and **f** we show the two-photon eigenspectra without and with disorder, respectively. Colors encode the two-photon eigenvalue ${\lambda }_{n,m}^{(2)}={\lambda }_{n}+{\lambda }_{m}$. For better appreciation we have separated the spectrum into the subspaces edge–edge $\left({\mathcal{E}}\otimes {\mathcal{E}}\right)$, bulk–edge $\left({\mathcal{B}}\otimes {\mathcal{E}}\right)$, and bulk–bulk $\left({\mathcal{B}}\otimes {\mathcal{B}}\right)$.

In optics, a first-order approximation of the Haldane model can be implemented using a honeycomb lattice composed of helical waveguides as illustrated in Fig. 1a, see pioneering work^41^. In this system, every waveguide has a nearest-neighbor coupling *κ*_1_ and a complex second-nearest-neighbor coupling *κ*_2_ or ${\kappa }_{2}^{* }$, see Fig. 1b. At the single-photon level, the Haldane lattice is governed by the Hamiltonian^1^

3 $$\hat{H}=\mathop{\sum} _{i}{\beta }_{i}{\hat{a}}_{i}^{\dagger }{\hat{a}}_{i}+{\kappa }_{1}\mathop{\sum} _{\langle i,j\rangle }\left({\hat{a}}_{i}^{\dagger }{\hat{a}}_{j}+{\hat{a}}_{j}^{\dagger }{\hat{a}}_{i}\right)+i{\kappa }_{2}\mathop{\sum} _{\langle \langle i,j\rangle \rangle }\left({\hat{a}}_{i}^{\dagger }{\hat{a}}_{j}-{\hat{a}}_{j}^{\dagger }{\hat{a}}_{i}\right),$$

where *β*_*i*_ represents the propagation constant of the *i*-th waveguide and the corresponding optical mode is represented by the creation (annihilation) operator, ${\hat{a}}_{i}^{\dagger }$ (${\hat{a}}_{i}$). Notice, in a disorder-free lattice *β*_*i*_ = *β*. The symbols 〈〉 and 〈〈〉〉 indicate summation over nearest and next-nearest-neighbor sites, respectively. The lattice used in our simulations is a ribbon with *N*_y_ = 90 hexagons in the *y*-direction and *N*_x_ = 10 hexagons in *x*-direction, Fig. 1c. We normalized the units in terms of *κ*_1_ throughout this work, and set *κ*_2_ = *iκ*_1_/5.

For pure states of two indistinguishable noninteracting particles the Hamiltonian is *H*_2_ = *H* ⊗ *I* + *I* ⊗ *H*, where *H* is the single-particle Hamiltonian and *I* is the identity operator^42^. The two-photon eigenstates are given by the symmetric tensor-product combinations of the single-photon eigenstates

4 $$|{\phi }_{m,n}^{(2)}\rangle =\left\{\begin{array}{l}\left|{\phi }_{m}\right\rangle \otimes \left|{\phi }_{n}\right\rangle \iff m=n,\hfill\\ \frac{1}{\sqrt{2}}\left(\left|{\phi }_{m}\right\rangle \otimes \left|{\phi }_{n}\right\rangle +\left|{\phi }_{n}\right\rangle \otimes \left|{\phi }_{m}\right\rangle \right)\iff m\,\ne\, n.\end{array}\right.$$

As alluded to above, the two-photon eigenvalues are the sums of the single-photon ones, ${\lambda }_{m,n}^{(2)}={\lambda }_{m}+{\lambda }_{n}$.

In the absence of disorder, the eigenvalue spectrum for single-photon states in a finite lattice exhibits topological edge states in the bandgap^43^, Fig. 1d. In contrast, for two indistinguishable photons, the spectrum does not have a bandgap: the edge–edge states can have the same eigenvalues ${\lambda }_{n,m}^{(2)}={\lambda }_{n}+{\lambda }_{m}$ as those lying in the bulk–bulk region, Fig. 1e.

To include disorder, we separate the lattice shown in Fig. 1c into three regions^1^. The left and right parts of the system are disorder-free, while its middle part exhibits diagonal disorder^31^, that is, random modifications of the on-site refractive index taken from a normal distribution with zero mean and variance *σ* = 1. Importantly, taking *σ* = 1 ensures that the disorder strength does not destroy the topological protection for single photons, since *σ* = 1 corresponds to half the size of the topological bandgap. The two-photon eigenspectrum in the presence of disorder is shown in Fig. 1f.

We now send trial two-photon wavepackets into the system. They are built from single-photon edge states and vary continuously from an unentangled product state, with Schmidt number *S*_N_ = 1, to highly entangled two-photon states, *S*_N_ ≫ 1^44,45^, with the two photons either correlated or anti-correlated in space^46^.

To construct these states, we begin with protected single-photon states as a template, $|{\tilde{\psi }}_{\sigma }^{(1)}\rangle =\mathop{\sum }\nolimits_{j = 1}^{{M}_{{\rm{e}}}}{(-1)}^{j}{e}^{-\frac{{\left({x}_{0}-j\right)}^{2}}{2{\sigma }^{2}}}\left|j\right\rangle$, where $\left|j\right\rangle$ describes a photon initialized in waveguide *j*, *M*_e_ = 20 is the selected range of waveguides in the upper-left edge of our system, and *x*_0_ = (*M*_e_ + 1)/2 = 10.5 is the center of this range. These single-photon wavepackets travel through both clean and disordered lattice without scattering to the bulk or back scattering. Importantly, the alternating sign (−1)^*j*^ in the amplitude ensures that the wavepacket has proper momentum and resides in the single-photon edge subspace.

Next, we construct our trial two-photon states as follows

5 $$|{\tilde{\psi }}_{{\sigma }_{{\rm{c}}},{\sigma }_{{\rm{a}}}}^{(2)}\rangle =\mathop{\sum }\limits_{j,k=1}^{{M}_{{\rm{e}}}}{\psi }_{j,k}\left|j,k\right\rangle =\mathop{\sum }\limits_{j,k=1}^{{M}_{{\rm{e}}}}{(-1)}^{j+k}{e}^{-\frac{{(j-k)}^{2}}{4{\sigma }_{{\rm{a}}}^{2}}-\frac{{\left({x}_{0}-(j+k)/2\right)}^{2}}{{\sigma }_{{\rm{c}}}^{2}}}\left|j,k\right\rangle .$$

Here, $\left|j,k\right\rangle$ represents the state where a photon starts at waveguide *j* and its twin at *k*. The spatial two-photon correlations are controlled by the parameters *σ*_c_ and *σ*_a_. For *σ*_c_ ≫ *σ*_a_ we have a spatially correlated state, in which both photons most probably enter into the same waveguide simultaneously^38^. For *σ*_a_ ≫ *σ*_c_ we obtain a spatially anti-correlated state, in which the two photons enter at two waveguides symmetrically lying on opposite sides of the window covered by the wavefunction^46^.

**Fig. 2:**
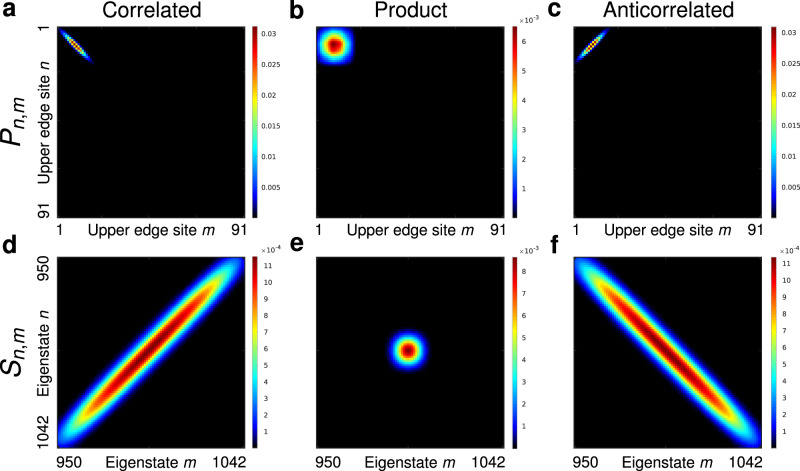
Initial two-photon states. **a**, **b**, **c** Spatial correlation maps *P*_*n*,*m*_ over the 91 sites of the upper edge of the lattice. **d**, **e**, **f** Spectral correlation maps *S*_*n*,*m*_ in the ${\mathcal{E}}\otimes {\mathcal{E}}$-subspace. **a**, **d** correspond to the strongly spatially correlated state $\left|{\psi }_{{\rm{c}}}^{(2)}\right\rangle$, $({\sigma }_{{\rm{c}}},{\sigma }_{{\rm{a}}})=(\sqrt{40},0.01)$, **b**, **e** stand for the product state $|{\psi }_{{\rm{p}}}^{(2)}\rangle$, $({\sigma }_{{\rm{c}}},{\sigma }_{{\rm{a}}})=(\sqrt{40},\sqrt{40})$, and **c**, **f** for the strongly spatially anti-correlated state $\left|{\psi }_{{\rm{a}}}^{(2)}\right\rangle$, $({\sigma }_{{\rm{c}}},{\sigma }_{{\rm{a}}})=(0.01,\sqrt{40})$.

Finally, we must ensure that the initial wavepackets only include edge states. To this end, we project our state onto the two-photon eigenstates $|{\phi }_{m,n}^{(2)}\rangle$ of the system and then remove the components belonging to the subspaces ${\mathcal{B}}\otimes {\mathcal{E}}$ and ${\mathcal{B}}\otimes {\mathcal{B}}$, keeping only states that belong to the edge–edge subspace

6 $$|{\psi }_{{\sigma }_{{\rm{c}}},{\sigma }_{{\rm{a}}}}^{(2)}\rangle =\frac{1}{A}\mathop{\sum }\limits_{m,n}^{{\mathcal{E}}\otimes {\mathcal{E}}}\mathop{\sum }\limits_{j,k=1}^{{M}_{{\rm{e}}}}{\psi }_{j,k}\langle {\phi }_{m,n}^{(2)}| j,k\rangle |{\phi }_{m,n}^{(2)}\rangle ,$$

where *A* is the normalization constant. It is worth noting that two-photon states described by Eq. (6) are a lattice adoption of Gaussian two-mode squeezed states^47^, which are a commonplace choice in quantum optical experiments. The corresponding spatial ${P}_{j,k}=| \langle j,k| {\psi }_{{\sigma }_{{\rm{c}}},{\sigma }_{{\rm{a}}}}^{(2)}\rangle {| }^{2}$ and spectral ${S}_{m,n}=| \langle {\phi }_{m,n}^{(2)}| {\psi }_{{\sigma }_{{\rm{c}}},{\sigma }_{{\rm{a}}}}^{(2)}\rangle {| }^{2}$ correlation maps of our initial states, Eq. (6), are shown in Fig. 2. Tuning *σ*_a_ and *σ*_c_, one can go from the spatially correlated state $|{\psi }_{{\rm{c}}}^{(2)}\rangle$, Fig. 2a, to the product state $|{\psi }_{{\rm{p}}}^{(2)}\rangle$, Fig. 2b, and to the spatially anti-correlated state $|{\psi }_{{\rm{a}}}^{(2)}\rangle$, Fig. 2c. Note the relation between spatial and spectral distributions: the state $|{\psi }_{{\rm{c}}}^{(2)}\rangle$, which is strongly correlated in space, Fig. 2a, is strongly anti-correlated spectrally Fig. 2d, and vice versa for $|{\psi }_{{\rm{a}}}^{(2)}\rangle$. Irrespective of their correlation maps, all these states occupy the same spatial area on the upper-left edge of the lattice, see Supplementary Note 1. The Schmidt number for $|{\psi }_{{\rm{c}},{\rm{a}}}^{(2)}\rangle$ is *S*_N_ = 13, while for $|{\psi }_{{\rm{p}}}^{(2)}\rangle$ we have *S*_N_ = 1. We now explore the robustness of these two-photon states as they traverse the disordered lattice. We begin with the product state $|{\psi }_{{\rm{p}}}^{(2)}\rangle$. To characterize the impact of disorder, we compute the fidelity^30^ that is given as the overlap of the state $|{\psi }_{{\rm{p}}}^{(2)}({z}_{{\rm{f}}})\rangle$ after it has traversed the lattice with the reference state $|{\psi }_{{\rm{p}}}^{(2)}({z}_{{\rm{m}}})\rangle$ obtained after propagating the same state $|{\psi }_{{\rm{p}}}^{(2)}\rangle$ in a disorder-free lattice, see Supplementary Note 2. The two wavepackets are taken at slightly different propagation distances *z*_f_ and *z*_m_ to account for the somewhat different travel distance in a disordered lattice. We find the fidelity ${F}_{{\rm{p}}}=| \langle {\psi }_{{\rm{p}}}^{(2)}({z}_{{\rm{f}}})| {\psi }_{{\rm{p}}}^{(2)}({z}_{{\rm{m}}})\rangle {| }^{2}=0.98$, confirming that both the single-photon states and their product are immune to disorder. The edge–mode content of the evolved state is almost 100%, ${E}_{{\rm{p}}}=\mathop{\sum }\nolimits_{n,m}^{{\mathcal{E}}\otimes {\mathcal{E}}}| \langle {\phi }_{n,m}^{(2)}| {\psi }_{{\rm{p}}}^{(2)}({z}_{{\rm{f}}})\rangle {| }^{2}=0.9934$. The product state traverses the lattice without distortion, see Supplementary Movie M1, in spite of the degeneracy between the two-photon edge–edge and bulk–bulk states.

**Fig. 3:**
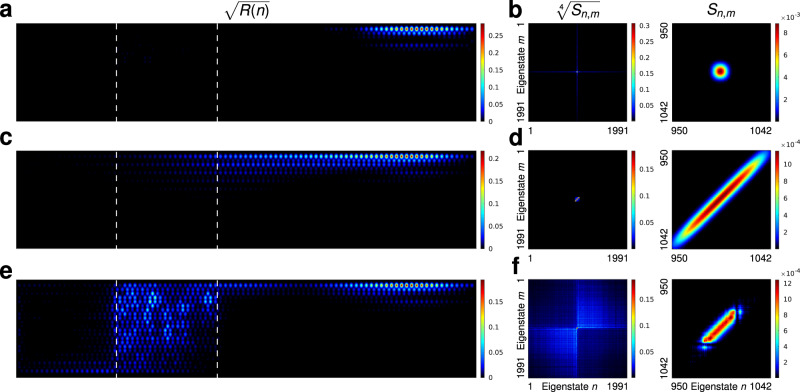
Propagation of two-photon edge states. **a** Probability density distribution for the reduced single-photon state and **b** the spectral correlation map for the product state $|{\psi }_{{\rm{p}}}^{(2)}\rangle$, which survives disorder. **c**, **d** The same for the spatially correlated entangled state $\left|{\psi }_{{\rm{c}}}^{(2)}\right\rangle$ propagating in the clean lattice, while **e**, **f** show the impact of disorder on this highly entangled state. In all cases the rightmost panels show a magnification of the edge–edge subspace. Dashed lines in **a**, **c**, **e** indicate the disordered region.

Figure 3a, b visualize this outcome by showing the single-photon spatial distribution and two-photon spectral correlation maps for the two-photon product state $|{\psi }_{{\rm{p}}}^{(2)}\rangle$ traversing the disordered lattice. The spatial single-photon probability density *R*(*n*) is given by the diagonal elements ${\rho }_{nn}^{(1)}$ of the reduced single-photon density matrix ${\hat{\rho }}^{(1)}$, $R(n)\equiv \left\langle n\right|{\hat{\rho }}^{(1)}\left|n\right\rangle \equiv {\rho }_{nn}^{(1)}$^48^. The reduced single-photon density matrix ${\hat{\rho }}^{(1)}$ is obtained from the two-photon density matrix ${\hat{\rho }}^{(2)}$ in the usual way, ${\hat{\rho }}^{(1)}=\mathop{\sum }\nolimits_{m}^{M}\left\langle m\right|{\hat{\rho }}^{(2)}\left|m\right\rangle$^30^. As expected, the spectral composition of the wavepacket remains undisturbed and the wavepacket propagates through the disordered region without leaving the edge.

We now turn our attention to entangled two-photon states. Figure 3c, d depict *R*(*n*) and the spectral correlation maps for the two-photon state $|{\psi }_{{\rm{c}}}^{(2)}\rangle$ traversing the “clean” lattice, and Fig. 3e, f show the same for the disordered lattice. While in the absence of disorder, *R*(*n*) stays on the edge and the highly correlated two-photon spectral distribution is unchanged (panels c, d), the disorder strongly affects these states. Figure 3e shows strong dissipation into the bulk as soon as the entangled wavepacket encounters the disordered region. The spectral distribution spreads all over the system, with both bulk–bulk and bulk–edge states becoming occupied, Fig. 3f. A similar result is obtained for $|{\psi }_{{\rm{a}}}^{(2)}\rangle$, except that the cross-like shape observed in Fig. 3f is flipped towards the opposite diagonal, Supplementary Note 3.

To quantify the probability fraction of the states scattered into the bulk we compute the edge–mode content. For $|{\psi }_{{\rm{c}}}^{(2)}\rangle$ the edge–mode content after traversing the disordered lattice is *E*_c_ = 0.4524, while for $|{\psi }_{{\rm{a}}}^{(2)}\rangle$ it gives *E*_a_ = 0.4453. Thus, >50% of both types of states is scattered into the bulk. The part of the states that survives the disordered region and stays on the edge remains strongly correlated in the spectral domain: the edge–edge part of its spectral content preserves the initial shape, see the right column in Fig. 3f. However, the spectral phase of the state is scrambled. To illustrate this point, we have renormalized the transmitted edge part of the two-photon wavepacket to unity and computed its fidelity *F*_N_ by overlapping it with the reference two-photon wavepacket from a clean system, yielding *F*_N_ = 0.405.

### The topological window of protection

**Fig. 4:**
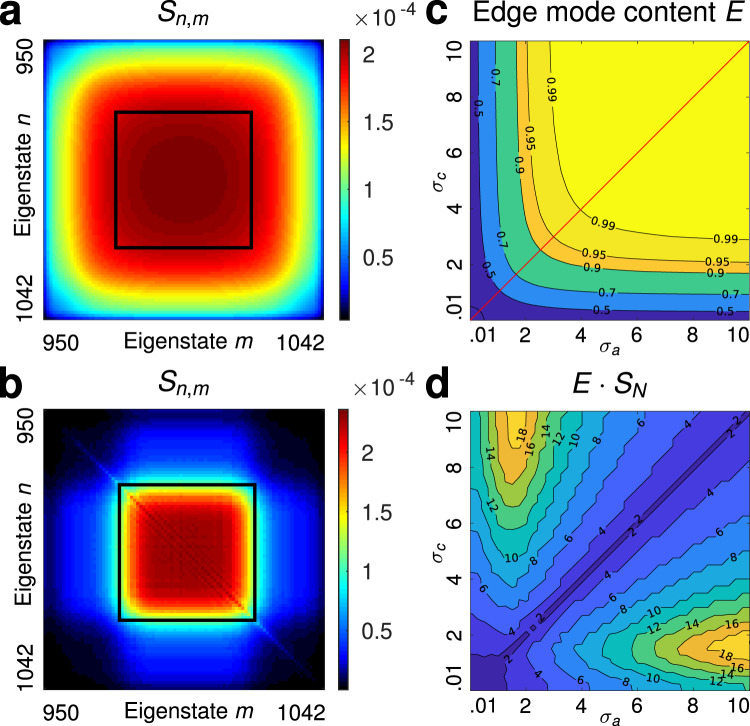
Topological window of protection. In order to identify the topological window of protection, we considered a spectrally broad product state with (*σ*_a_ = *σ*_c_ = 0.01) as initial state and propagate it through an ensemble of 1000 random Haldane lattices. In **a** we depict the spectral correlation map for the initial state. **b** depicts the ensemble-average of the spectral correlation maps inside the edge–edge subspace after the propagation through the ensemble of disordered lattices. We find that the only two-photon amplitudes that survive the disorder lie in the region indicated by the black square, which is the protection window. **c** shows the edge–mode content E and **d** the product of the edge–mode content and the Schmidt-number *E* ⋅ *S*_*N*_ as a function of the parameters *σ*_a_, *σ*_c_ of the initial states in the range *σ*_a_, *σ*_c_ ∈ [0.01, 10].

We find that the conduit for dissipation of the two-photon edge–edge states is always provided by the edge–bulk states, which are degenerate in energy with the edge–edge states. Once disorder induces transitions into the edge–bulk states, they further transfer the amplitudes into the energy-degenerate bulk–bulk states, see Supplementary Movies M2 and M3. Hence, the key to topological protection is to minimize the disorder-induced overlap of the initial joint spectrum with the edge–bulk and bulk–bulk spectral regions, keeping it as close to the center as possible. That is, there is a topological protection window for two-photon states that offers the key guideline for designing robust two-photon states. To infer the protection window, we sent a probe product state with *σ*_c_ = *σ*_a_ = 0.01 through an ensemble of 1000 disordered lattices. This initial state is very well localized onto the edge region in real space, ensuring that all components within the state travel along very close paths. The spectral content of the state before and after the disorder is shown in in Fig. 4a, b. The components that have survived the impact of disorder are within the marked window—the topological window of protection. The joint spectral correlation map of any entangled state with varying *σ*_a_ and *σ*_c_ must fit inside this protection window to be robust against disorder.

In practice, to increase the amount of entanglement we need to increase *σ*_a_$\left({\sigma }_{{\rm{c}}}\right)$ while decreasing *σ*_c_$\left({\sigma }_{{\rm{a}}}\right)$, and by doing so the joint spectrum unavoidably tends to fall outside the protection window. However, we can always find two-photon states with a considerable amount of entanglement, which are protected. To elucidate this we have scanned the edge-mode content of the two-photon states after propagation through the disordered region as a function of *σ*_a_ and *σ*_c_. In Fig. 4c, d we show the contour maps of the edge–mode content as we vary *σ*_a_ and *σ*_c_. Figure 4c shows the edge–mode content of the two-photon states after propagation through the disordered region, with the diagonal corresponding to the product states, that is, states with *σ*_a_ = *σ*_c_. The states with the highest degree of entanglement correspond to very different *σ*_a_ and *σ*_c_ and, therefore, they are found in the top left and lower right corners in Fig. 4c. In general, highly entangled states lay on the top left $\left({\sigma }_{{\rm{a}}}\ll {\sigma }_{{\rm{c}}}\right)$ and bottom right $\left({\sigma }_{{\rm{a}}}\gg {\sigma }_{{\rm{c}}}\right)$ corners and the edge–mode content quickly drops below 0.5. The reason is because as one increases *σ*_a_, or *σ*_c_, the tails of the spectral correlation ellipse fall outside of the protection window and, as a result, the states scatter into the bulk. Similarly, uncorrelated states may experience the same fate when they are initially confined into a small spatial region, which is the case for states with ${\sigma }_{{\rm{a}}}={\sigma }_{{\rm{c}}}\in \left(0,2.5\right)$. Figure 4d shows the key figure of merit, *E* ⋅ *S*_N_, the product of the Schmidt number *S*_N_ and the edge–mode content *E*. The bright yellow islands indicate the best two-photon states, which combine robustness against disorder with high degree of entanglement. Importantly, the spectral correlation ellipse of these states always fits into the protection window shown in Fig. 4b. It is worth mentioning the features exhibited by the contour maps are generic as similar structures are obtained for disordered Haldane lattices with different dimensions, see Supplementary Note 4. This demonstrates that, in principle, one can create states with high Schmidt number and edge–mode content close to unity.

As evidence that our results are generic, in the sense that they apply to other two-dimensional topological systems, in the Supplementary Note 5 we have performed a similar analysis for an aperiodic topological lattice system^6,40^. We have found that the contour map of the edge-mode content *E* is not symmetric, implying that the correlated states are slightly less protected than their anti-correlated mirror-images. Nevertheless, we obtain the same qualitative features as in the Haldane model.

## Discussion

Before concluding, we would like to outline possible ways to generate the initial states and address the potential challenges for experimental observations of these effects. The initially highly correlated states can be implemented using standard spontaneous-parametric-down-conversion nonlinear crystals to generate photon pairs that are coupled to the edge of the lattice using a positive achromatic doublet lens as demonstrated in^38^. Anti-correlated photon pairs can be generated by applying the fractional Fourier transform to the highly correlated states^49^. The Haldane lattice has been previously demonstrated using femtosecond laser written waveguides as reported in^41^. Hence, the challenges are reduced to optimizing the fabrication for minimal scattering, absorption and bending losses associated with the helical waveguides.

These results lead to the following conclusions. Two issues have to be considered when constructing two-photon entangled edge states in topological systems: their dissipation into the bulk and the relative dephasing between the different components comprising the entangled state. Regarding dissipation, the two-photon edge states can be protected just as well as the single-photon edge states. Further, phase scrambling can also be minimized if the different components of the entangled state travel along the same path in the edge region. Both aims are achieved by keeping the spectral correlation map of the two-photon state in the center of the window of protection. Thus, attempts to increase entanglement must be balanced against keeping the spectral correlation maps of the two-photon states within the narrow spectral region at the very center of the single-photon gap—the topological window of protection. This limits the degree of entanglement one can safely encode in practice, but presents a clear strategy for creating useful states with high degree of entanglement and robustness.

Looking forward, one could take advantage of the static nature of disorder to circumvent entanglement-induced dissipation into the bulk. While the disorder-induced relative phase between the different product-state components of the entangled wavepacket may appear random due to the random nature of disorder, for static disorder scrambling and dissipation are nevertheless fixed. This opens an opportunity to find the windows of protection as we have done in the cases considered here, and generate robust wavepackets tailored to the particular disordered system at hand. From a practical perspective, the stability of entangled states up to relatively high Schmidt numbers offers practical guidelines for generating useful entangled edge states in topological photonic systems. Finally, our work may open the door to study topological protection of highly entangled multiphoton non-Gaussian states that fulfill the protection conditions.

## Supplementary information

Supplementary Information

Description of Additional Supplementary Files

Supplementary Movie 1

Supplementary Movie 2

Supplementary Movie 3

Supplementary Movie 4

Supplementary Movie 5

## Data Availability

The data that support the findings of this study are available from the corresponding author upon reasonable request.

## Code availability

The Matlab programs used to simulate the systems discussed in the paper are available as supplementary software.
