# Supplementary material for: Topological protection versus degree of entanglement of two-photon light in photonic topological insulators
Source: Nat Commun. 2021 Mar 30;12:1974. doi: 10.1038/s41467-021-22264-3 (PMC8009886; doi:10.1038/s41467-021-22264-3)
Supplement: Supplementary file 1 — Supplementary Information [file 41467_2021_22264_MOESM1_ESM.pdf]

Supplementary Information

Topological protection versus degree of entanglement of two-photon light in photonic topological insulators

Konrad Tschernig,[1,2,*] Álvaro Jimenez-Galán,[1] Demetrios N. Christodoulides,[3]
Misha Ivanov,[1,2,4] Kurt Busch,[1,2] Miguel A. Bandres,[3,†] and Armando Perez-Leija[1,2,‡]

[1]*Max-Born-Institut, Max-Born-Straße 2A, 12489 Berlin, Germany*

[2]*Humboldt-Universität zu Berlin, Institut für Physik,*
AG Theoretische Optik & Photonik, Newtonstraße 15, 12489 Berlin, Germany

[3]*CREOL, The College of Optics and Photonics,*
University of Central Florida, , Orlando, FL 32816-2700, USA

[4]*Blackett Laboratory, Imperial College London, London, UK*

This document is structured as follows: In section I we present a more detailed description of the construction of the initial states. In sections II and III we discuss the propagation of the two-photon states in the clean system and the disordered lattices, respectively. In section IV we discuss the effects of the lattice size on the protection window. Finally, in section V we describe the analysis of the impact of disorder on highly-entangled two-photon states propagating in the aperiodic topological insulator.

SUPPLEMENTARY NOTE 1 - CONSTRUCTION OF THE INITIAL TWO-PHOTON STATES

As explained in the main text, we constructed the initial states by choosing different values for σ_c and σ_a in the expression

$$|\tilde{\psi}^{(2)}_{\sigma_c,\sigma_a}\rangle = \sum_{j,k=1}^{M_e} \psi_{j,k}\,|j,k\rangle = \sum_{j,k=1}^{M_e} (-1)^{j+k} e^{-\frac{(j-k)^2}{4\sigma_a^2}-\frac{(x_0-(j+k)/2)^2}{\sigma_c^2}}\,|j,k\rangle\,, \tag{1}$$

which we then project onto the $\mathcal{E}\otimes\mathcal{E}$-subspace and renormalize the resulting state. As examples, we obtain the correlated state $|\psi^{(2)}_c\rangle$ ($\sigma_c = \sqrt{40}$, $\sigma_a = 0.01$), the semi-correlated state $|\psi^{(2)}_{sc}\rangle$ ($\sigma_c = \sqrt{40}$, $\sigma_a = \sqrt{40}/3$), the product state $|\psi^{(2)}_p\rangle$ ($\sigma_c = \sqrt{40}$, $\sigma_a = \sqrt{40}$), the semi-anticorrelated state $|\psi^{(2)}_{sa}\rangle$ ($\sigma_c = \sqrt{40}/3$, $\sigma_a = \sqrt{40}$) and finally the anti-correlated state $|\psi^{(2)}_a\rangle$ ($\sigma_c = 0.01$, $\sigma_a = \sqrt{40}$). In Fig. (1) we show the spatial and spectral correlation maps as well as the reduced density matrix representations of these states. As one can see, despite the fundamentally different correlation maps, all states occupy the same spatial region on the upper left edge of the lattice.

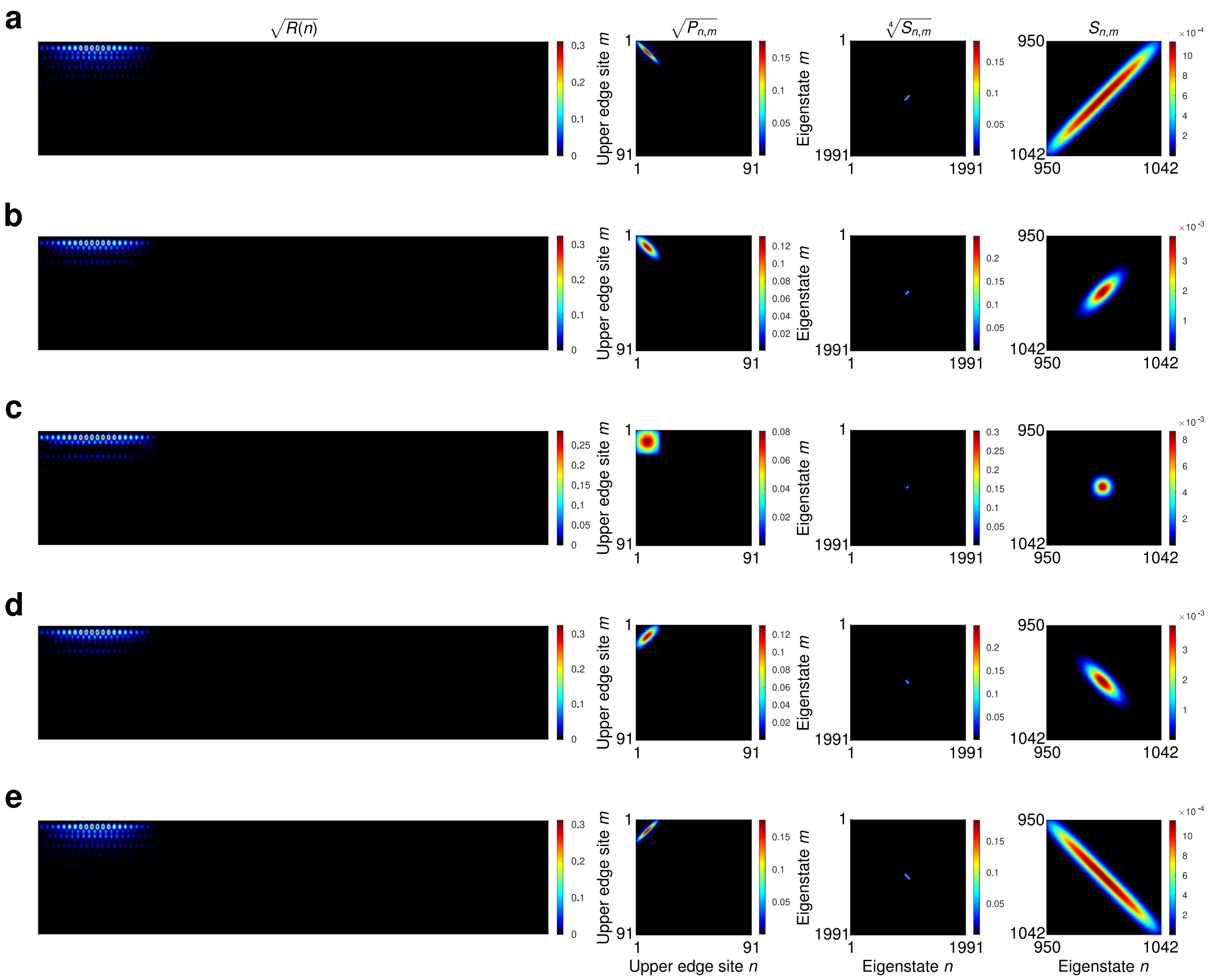

Figure 1: **Reduced density matrix representation $R(n)$, the spatial correlation map $P_{n,m}$, the spectral correlation map $S_{n,m}$ for the five different initial states considered in our simulations.** **a** Correlated state, **b** semi-correlated state, **c** correlated state, **d** semi-anticorrelated state and **e** anticorrelated state. Note that we show the square-root/fourth-root - as indicated above the panels - in order to increase the visibility of components with small probability.

SUPPLEMENTARY NOTE 2 - PROPAGATION IN THE CLEAN SYSTEM

In order to assess the impact of disorder on two-photon states, we first analyze their propagation in a disorder-free lattice. Fig. (2) depicts the final states after a propagation distance $z_c = 75$. Even though there is no disorder present in the system, in the two cases, $|\psi_c^{(2)}\rangle$ and $|\psi_a^{(2)}\rangle$, the spatial probability distribution tends to spread out over the edge of the lattice losing the initial Gaussian shape, as illustrated in Figs. (2 a-e). In terms of correlations, both states spread out towards the four corners of the spatial correlation map. However, as the spreading is more prominent along the main diagonal we assert that in both cases the photons tend to bunch into the same site. Concurrently, we observe the emergence of interference fringes parallel (orthogonal) to the main diagonal of the correlation map corresponding to the correlated (anticorrelated) state. This implies that certain states are suppressed as a result of destructive quantum interference. Naturally, the spectral correlation maps remain invariant upon propagation, as well as the edge-mode content $E_p = 1$ ensuring that only edge modes are

present in the evolved wave-packet.

It is important to stress that the broadening of the wavefunctions is due to multimode

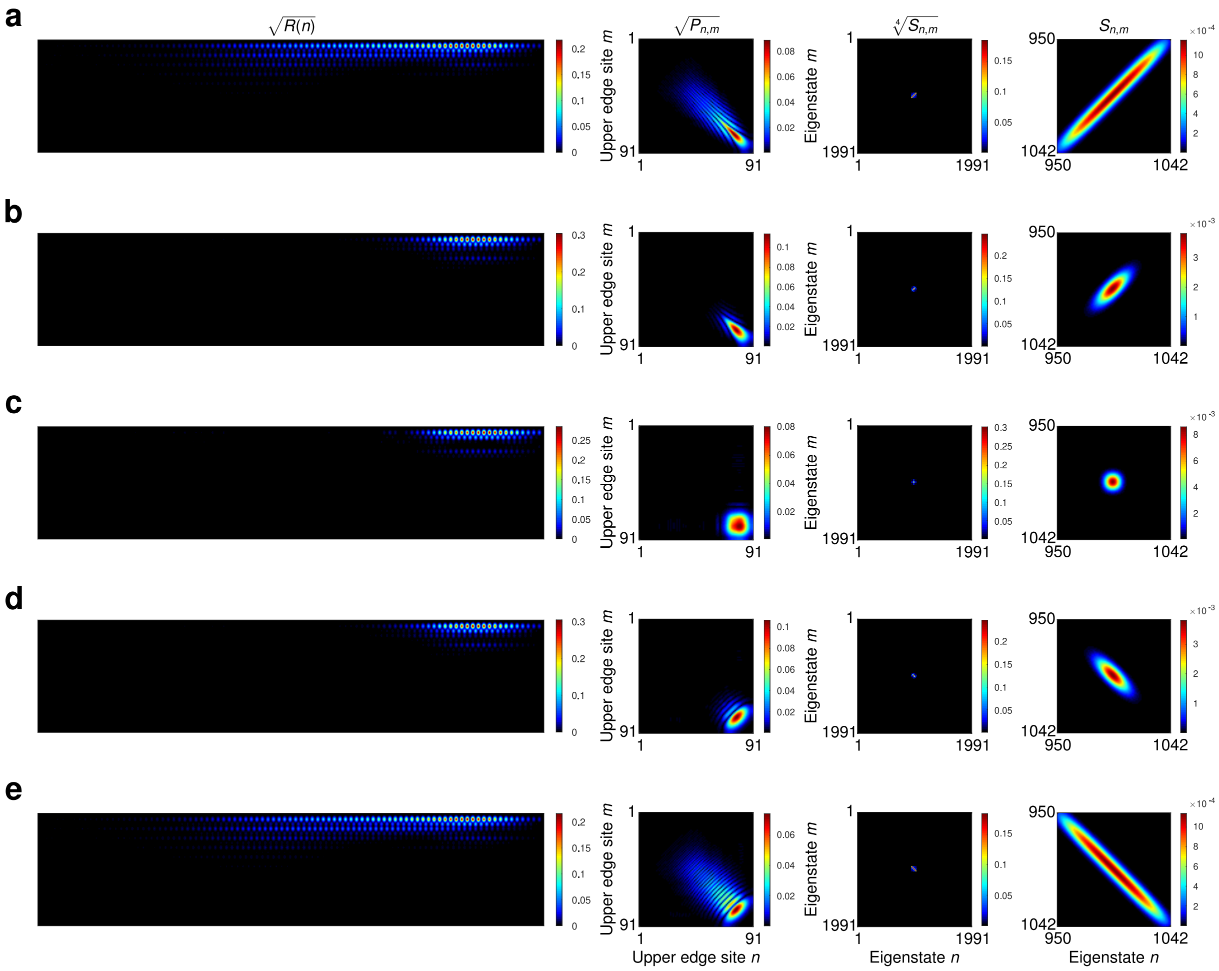

Figure 2: **Reduced density matrix $R(n)$, spatial $P_{n,m}$, and spectral $S_{n,m}$ correlation maps for the five states considered in our simulations after propagation distance $z_c = 75$ in the clean lattice.** **a** Correlated state, **b** semi-correlated state, **c** correlated state, **d** semi-anticorrelated state and **e** anticorrelated state. Note that we show the square-root/fourth-root - as indicated above the panels - in order to increase the visibility of components with small probability.

interference, and since the two-photon eigenmodes exhibit larger propagation eigenvalues, the spreading rate is faster compared to single-photon wavepackets. In view of the spatial distortions undergone by $|\psi_c^{(2)}\rangle$ and $|\psi_a^{(2)}\rangle$, one can directly state that entangled states degrade even in disorder-free Haldane topological lattices. This intrinsic dispersion of strongly entangled states - even without disorder - may pose an additional challenge for their application in topological quantum information processing.

SUPPLEMENTARY NOTE 3 - PROPAGATION THROUGH DISORDERED LATTICES

Here we present the resulting states after the propagation through disorder. The correlated $|\psi_c^{(2)}\rangle$ and anticorrelated $|\psi_a^{(2)}\rangle$ two-photon states scatter significantly into the bulk of the disordered region. In the first place, spatial correlations, Figs. (3-a) and (3-e), present some notable

differences with their counterparts obtained in the clean system, Figs. (2-a) and (2-e). That is, in the disordered cases the correlation maps no longer broaden along the main diagonal but they expand away of it redistributing the probabilities into three lobes. Yet, the highest probabilities are localized in the central lobe indicating that the photons remain mainly correlated and anticorrelated as the corresponding initial states. Accordingly, in the spectrum the wavefunctions turn to be wider as demonstrated by the correlation maps shown in the third columns of Figs. (3-a) and (3e). A closer look into the central probability lobes, shown in the right-most column, reveals that the spectrally anticorrelated and correlated nature of the initial states survive the impact of disorder to some extent. Indeed, by monitoring the full dynamics one can see how the wavefunctions lose their correlation properties upon scattering and eventually the transmitted parts recover the initial correlation structure. We provide simulations of the complete scattering process for all 5 different states in the supplementary movies, M1 to M5.

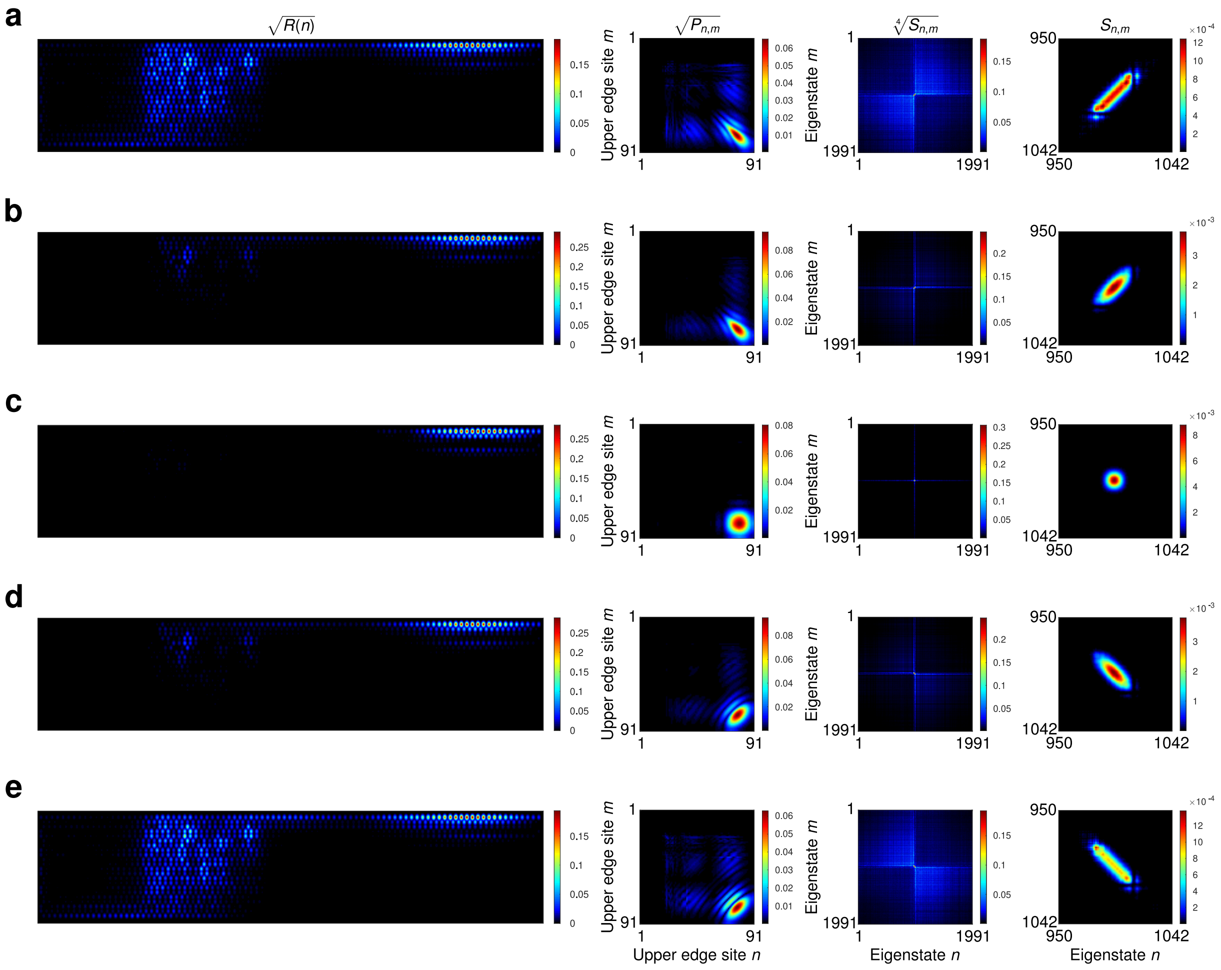

Figure 3: **Reduced density matrix $R(n)$, the spatial $P_{n,m}$, and the spectral $S_{n,m}$ correlation maps for the five different states considered in our simulations after propagation distance $z_d = 78.5$ in the disordered lattice.** **a** Correlated state, **b** semi-correlated state, **c** correlated state, **d** semi-anticorrelated state and **e** anticorrelated state. Note that we show the square-root/fourth-root - as indicated above the panels - in order to increase the visibility of components with small probability.

SUPPLEMENTARY NOTE 4 - EFFECTS OF THE LATTICE SIZE

We now explore how the size of the Haldane lattice influences the protection window. To do so, we consider two additional lattices with double spatial length ($N_x = 10, N_y = 180$ hexagons) and width ($N_x = 20, N_y = 90$). In both cases the length of the disordered region ($N_d = 20$ hexagons in y-direction) is the same as in the original lattice ($N_x = 10, N_y = 90$), see Fig. (4). The parameter scans in Fig. (5) yield, essentially, the same contour maps for the edge-mode content E and the product $E \cdot S_N$. To further corroborate this finding, we tested even larger systems ($N_x = 90, N_y = 10, 20, 40, 60, 80$), for a correlated state with $\sigma_a = 0.01$ and $\sigma_c = 5$. As depicted in Fig. (6), the edge-mode content of this state, after the disordered region, is also independent of the system size. Accordingly, we conclude that the results discussed in the main text are generic and not a mere effect of the system size.

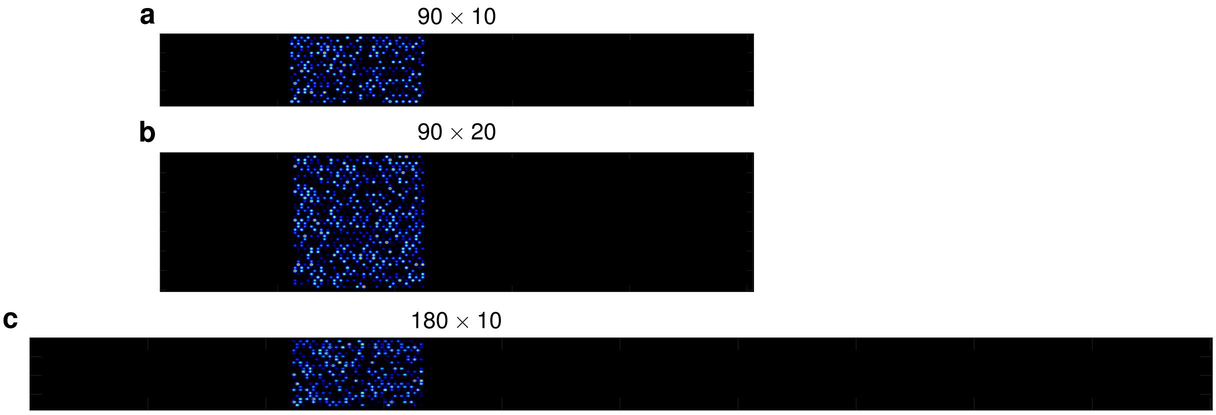

Figure 4: **Sketch of the lattices considered in the analysis of the impact of the system size.** **a** Original lattice from the manuscript. **b** Lattice with twice the width. **c** Lattice with twice the length. In all cases, the disordered region has the same length $N_d = 20$.

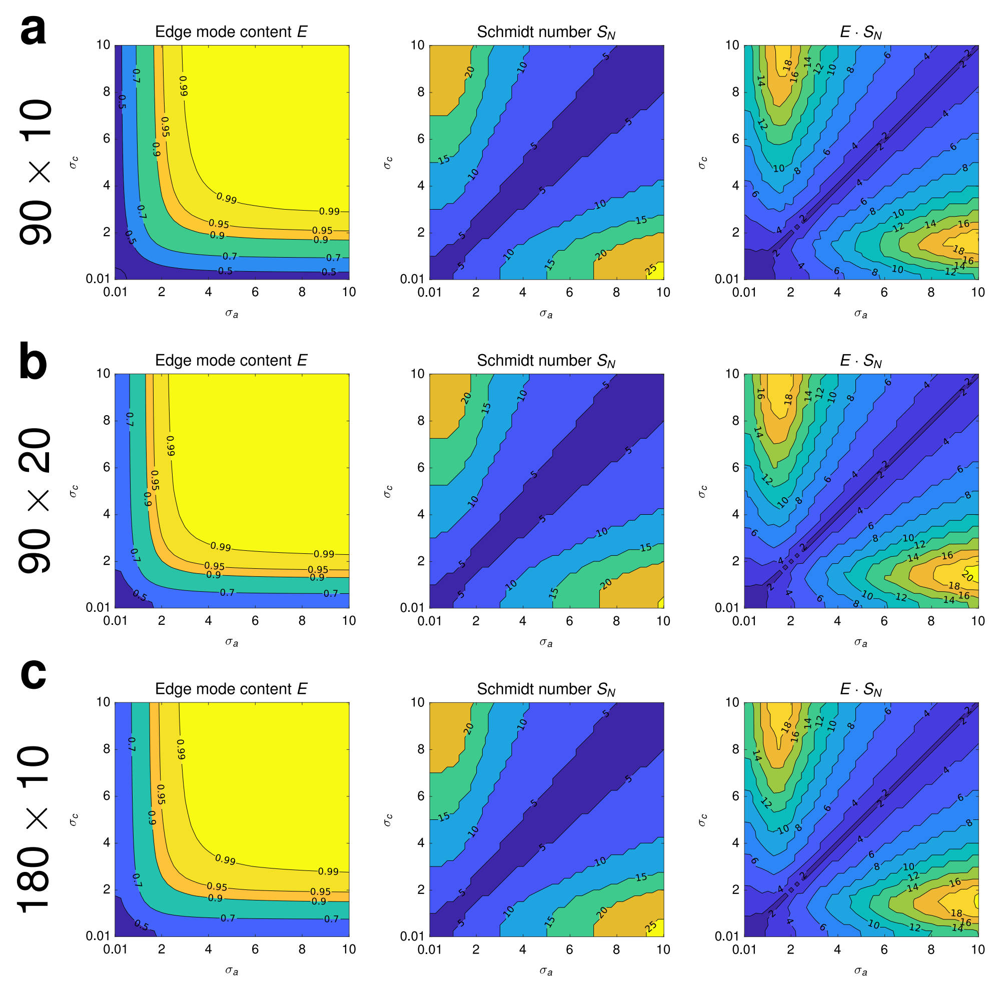

Figure 5: **Results of the parameter scans of** (σ_a, σ_c)**.** The columns correspond to (from the left to the right) the edge-mode content E, Schmidt number S_N and the combined figure of merit $E \cdot S_N$. **a** Original lattice. **b** Double width. **c** Double length. All contour maps display the same features, where highly entangled states (close to the σ_a-/σ_c-axis) are highly impacted by disorder.

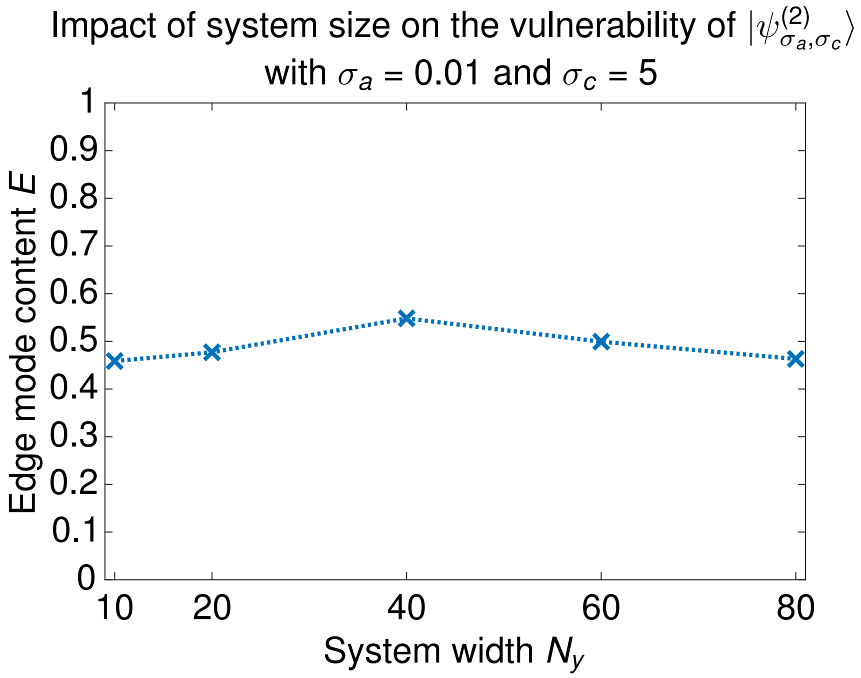

Figure 6: **Further analysis of the impact of system size.** Plot of the edge-mode content E of the correlated state $|\psi^{(2)}_{\sigma_a,\sigma_c}\rangle$ after the disorder, with $\sigma_a = 0.01$, $\sigma_c = 5$, against increasing widths of the lattice $N_x = 90, N_y = 10, 20, 40, 60, 80$. As one can see, E remains close to 0.5 in all cases, as a result, we conclude that the impact of disorder on highly entangled states is independent of the lattice size.

SUPPLEMENTARY NOTE 5 - TOPOLOGICAL PROTECTION OF ENTANGLED TWO-PHOTON STATES IN AN APERIODIC TOPOLOGICAL INSULATOR

In order to show that our results are applicable to other 2D topological systems - and to connect them with established experimental work in photonic systems, we now consider the evolution of two-photon states in a topological insulator based on an aperiodic lattice. Such a system has been implemented experimentally using aperiodic networks of coupled ring-resonators[1–3]. At the single-particle level, this system is described by the Hamiltonian

$$\hat{H} = \kappa \sum_{n,m} \hat{a}^{\dagger}_{n,m}\hat{a}_{n,m+1} + e^{-i\phi m}\hat{a}^{\dagger}_{n,m}\hat{a}_{n+1,m} + h.c. \tag{2}$$

The site modes represented by the operators $\hat{a}^{\dagger}_{n,m}$ form a 2D-square lattice with nearest-neighbor hopping, where the coupling κ in y-direction (left to right edge, index m) is real-valued. In the x-direction (top to bottom edge, index n) the sites exhibit a complex-valued coupling $\kappa e^{-i\phi m}$, which is dependent on the y-coordinate m, as sketched in Fig. (7-a). Specifically we choose $\phi = \frac{\pi}{2}$, which ensures that the phase accumulated around any local 4-site plaquette is $-\phi m + \phi(m+1) = \phi = \frac{\pi}{2}$. For our simulations we consider a finite ribbon with $N_x \times N_y = 20 \times 90$ sites, Fig. (7-b), where the vertical red lines indicate the region where we introduce static disorder. The single-photon spectrum (without disorder) features two disjoint edge-spaces $\mathcal{E}_{\pm}$, which correspond to clock-wise (CW, $\mathcal{E}_{+}$) and counter-clockwise (CCW, $\mathcal{E}_{-}$) propagating edge-modes, Fig. (7-c). As we have done for the Haldane lattice, we prepare states that start on the top-left edge of the system. As such, it is convenient to project them only onto the $\mathcal{E}_{-}$-subspace, where the states then propagate CCW directly into the disordered region. Thus we define the $\mathcal{E}_{+}$-space to be part of the bulk space $\mathcal{B}$, which ensures that - despite dissipation effects - also back-scattering is reflected in the edge-mode content of the states after propagation through the disorder. We show the resulting two-photon spectrum (without disorder) in Fig. (7-d), which indicates again the massive degeneracies between the $\mathcal{B}\otimes\mathcal{B}$, $\mathcal{B}\otimes\mathcal{E}$ and $\mathcal{E}\otimes\mathcal{E}$ two-photon subspaces.

We construct the two-photon states in the same way as for the Haldane lattice using the template states

$$|\psi^{(2)}_{\sigma_c,\sigma_a}\rangle = \sum_{j,k=1}^{M_e} (-1)^{j+k} e^{-\frac{(j-k)^2}{4\sigma_a^2} - \frac{(x_0-(j+k))^2}{\sigma_c^2}} |j,k\rangle \,. \tag{3}$$

After projection onto the $\mathcal{E}\otimes\mathcal{E}$-subspace, and renormalization, we obtain the exemplary states shown in Fig. (8). In comparison to the Haldane lattice, these states display very similar spatial correlations (by construction) but slightly different spectral correlation maps. Specifically, their spectral correlation ellipses are not oriented around the center of the edge-edge subspace. This is a consequence of the aperiodic nature of the lattice, which induces an asymmetric dispersion

relation with respect to the center of the $\mathcal{E}_-$ ($\mathcal{E}_+$) subspace in the single-photon spectrum, Fig. (7-c). However, we still observe that the spatially correlated state is spectrally anti-correlated, and vice versa for the spatially anti-correlated state.

In order to identify the window of protection, we launch the spectrally wide product-state $|\psi^{(2)}_{\sigma_c=0.5,\sigma_a=0.5}\rangle$ through an ensemble of 200 instances of disordered lattices (strength of the disorder $\sigma = 0.3$) and observe the surviving spectral amplitudes in Fig. (9). Notably, also the window of protection does not lie in the center of the edge-edge subspace. However, we can deduce that more highly entangled states will be less protected in this system. This is indeed the case, as one can see in the parameter-scans over (σ_c, σ_a) in Fig. (10). In complete analogy to the Haldane lattice, the most strongly entangled states - largest Schmidt-number S_N - are close to the σ_a-/σ_c-axes, where also the edge-mode content E after propagation through the disorder is the lowest. Quite interestingly, we observe an asymmetry between correlated- and anti-correlated states, such that correlated states ($\sigma_a > \sigma_c$) are protected to a lesser degree than their anti-correlated "mirror images" ($\sigma_a < \sigma_c$). We conjecture, that this is a consequence of the aperiodicity of the lattice as well. We stress that the dispersion of the two-photon wavepackets is significantly stronger in comparison to the Haldane lattice. The reduced single-photon state spreads around the complete edge of the system even before the slower parts of the wavefunction can leave the disordered region. This poses a challenge to the comparability of the results. But nevertheless, we observe very similar features and conclude that our results also apply in the present aperiodic topological system.

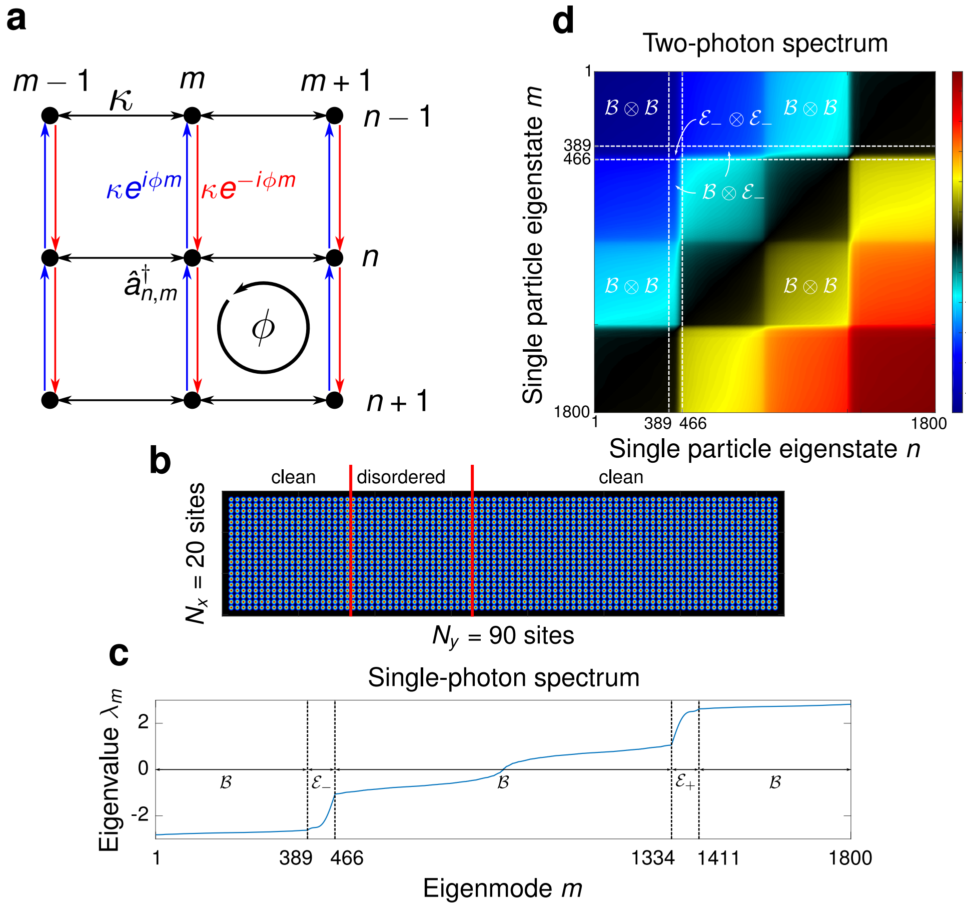

Figure 7: **Aperiodic topological lattice.** **a** Sketch of the coupling structure in the aperiodic topological insulator. **b** Finite ribbon considered in our simulations. **c** Single-photon spectrum. **d** Two-photon spectrum.

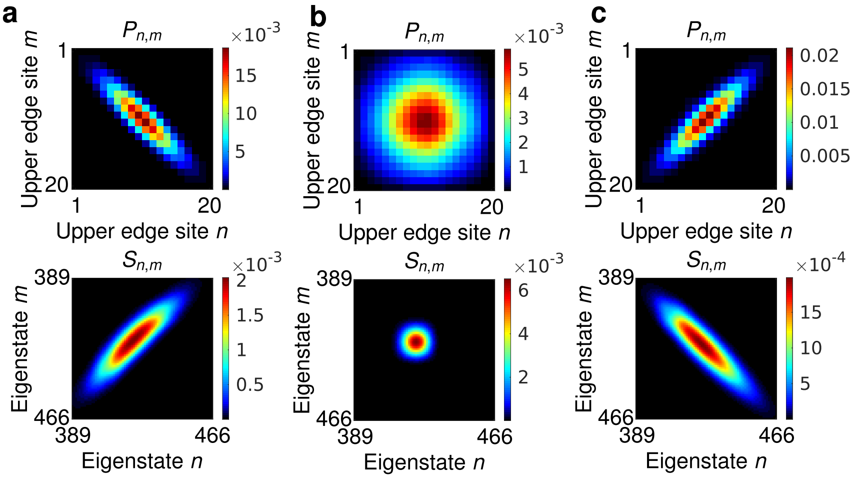

Figure 8: **Spatial $P_{n,m}$ and spectral $S_{n,m}$ correlation maps in the aperiodic lattice.** **a** Spatially correlated state with $\sigma_a = 1.2, \sigma_c = 6$. **b** Product state with $\sigma_a = \sigma_c = 6$. **c** Spatially anti-correlated state with $\sigma_a = 6, \sigma_c = 1.2$.

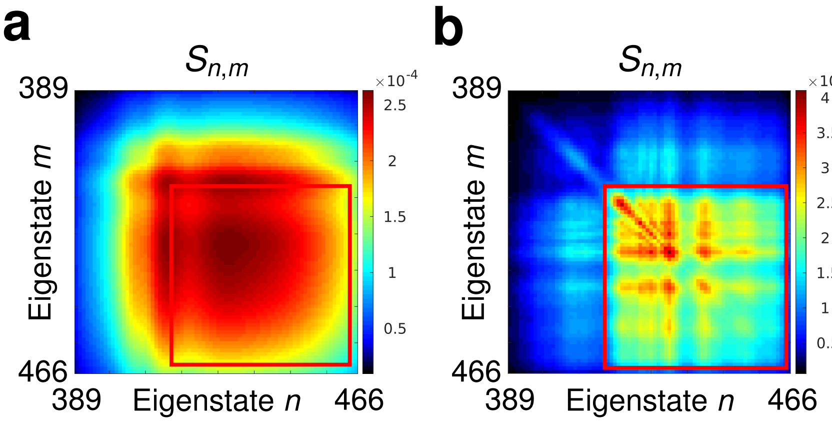

Figure 9: **Protection window in the aperiodic lattice.** **a** Initial state to probe the window of protection, which is product state with $\sigma_a = 0.5 = \sigma_c$. **b** Ensemble average spectral correlation map after propagation through 200 instances of the random disorder. The window of protection is marked by the red square.

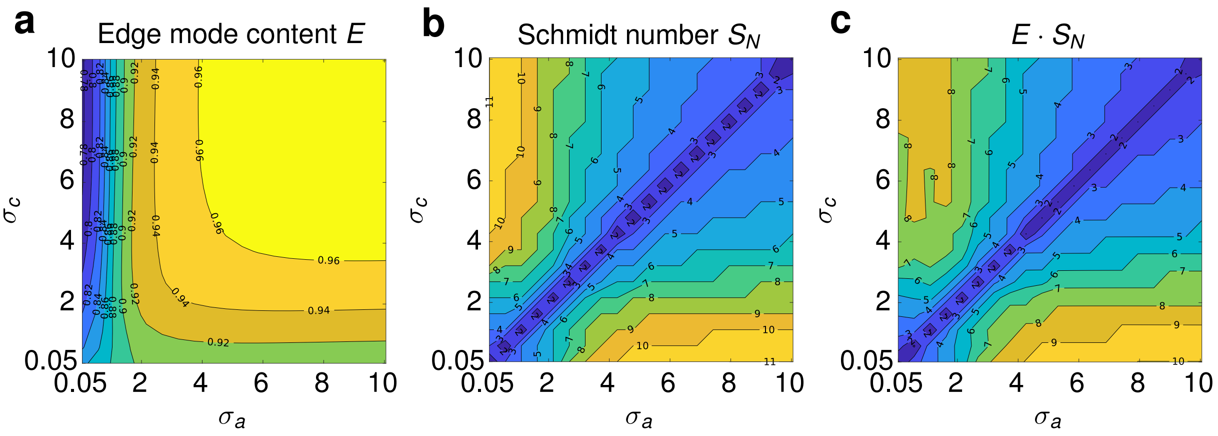

Figure 10: **Parameter scans over the entanglement parameters σ_a and σ_c.** **a** Contour-plot of the edge-mode content E after propagation through the disordered aperiodic lattice (disorder strength $\sigma = 0.3$, propagation distance is $z = 175$). **b** Schmidt-numbers S_N of the initial states. **c** The figure of merit $E \cdot S_N$.

* Electronic address: konrad.tschernig@physik.hu-berlin.de

† Electronic address: bandres@creol.ucf.edu

‡ Electronic address: apleija@gmail.com

[1] M. Hafezi, E. A. Demler, M. D. Lukin, and J. M. Taylor, Nature Physics 7, 907 (2011).

[2] M. Hafezi, S. Mittal, J. Fan, A. Migdall, and J. M. Taylor, Nature Photonics 7, 1001 (2013).

[3] G. Harari, M. A. Bandres, Y. Lumer, M. C. Rechtsman, Y. D. Chong, M. Khajavikhan, D. N. Christodoulides, and M. Segev, Science 359 (2018).
