# Supplementary material for: Topological protection versus degree of entanglement of two-photon light in photonic topological insulators
Source: Nat Commun. 2021 Mar 30;12:1974. doi: 10.1038/s41467-021-22264-3 (PMC8009886; doi:10.1038/s41467-021-22264-3)
Supplement: Supplementary file 2 — Description of Additional Supplementary Files [file 41467_2021_22264_MOESM2_ESM.pdf]

Description of Additional Supplementary Files

Title: Supplementary Movie 1

Description: Propagation of the product state through a disordered Haldane lattice

Title: Supplementary Movie 2

Description: Propagation of the correlated entangled state through the disordered Haldane lattice

Title: Supplementary Movie 3

Description: Propagation of the anticorrelated entangled state through the disordered Haldane lattice

Title: Supplementary Movie 4

Description: Propagation of the semi-anticorrelated entangled state through the disordered Haldane lattice

Title: Supplementary Movie 5

Description: Propagation of the semi-correlated entangled state through the disordered Haldane lattice

Title: Supplementary Code

Description: Code package to perform the simulations and reproduce the figures presented in the paper and supplementary information. Note, it requires matlab to execute.
